# TIF-Reg: Point Cloud Registration with Transform-Invariant Features in SE(3)

**DOI:** 10.3390/s21175778

**Published:** 2021-08-27

**Authors:** Baifan Chen, Hong Chen, Baojun Song, Grace Gong

**Affiliations:** 1 School of Automation, Central South University, Changsha 410083, China; hong.c@csu.edu.cn (H.C.); songbaojun@csu.edu.cn (B.S.); 2 The Cheriton School of Computer Science, University of Waterloo, Waterloo, ON N2L 3G1, Canada; gracegn@uwaterloo.ca

**Keywords:** transform-invariant feature, point cloud, registration

## Abstract

Three-dimensional point cloud registration (PCReg) has a wide range of applications in computer vision, 3D reconstruction and medical fields. Although numerous advances have been achieved in the field of point cloud registration in recent years, large-scale rigid transformation is a problem that most algorithms still cannot effectively handle. To solve this problem, we propose a point cloud registration method based on learning and transform-invariant features (TIF-Reg). Our algorithm includes four modules, which are the transform-invariant feature extraction module, deep feature embedding module, corresponding point generation module and decoupled singular value decomposition (SVD) module. In the transform-invariant feature extraction module, we design TIF in SE(3) (which means the 3D rigid transformation space) which contains a triangular feature and local density feature for points. It fully exploits the transformation invariance of point clouds, making the algorithm highly robust to rigid transformation. The deep feature embedding module embeds TIF into a high-dimension space using a deep neural network, further improving the expression ability of features. The corresponding point cloud is generated using an attention mechanism in the corresponding point generation module, and the final transformation for registration is calculated in the decoupled SVD module. In an experiment, we first train and evaluate the TIF-Reg method on the ModelNet40 dataset. The results show that our method keeps the root mean squared error (RMSE) of rotation within 0.5${}^{\circ }$ and the RMSE of translation error close to 0 m, even when the rotation is up to [−180${}^{\circ }$, 180${}^{\circ }$] or the translation is up to [−20 m, 20 m]. We also test the generalization of our method on the TUM3D dataset using the model trained on Modelnet40. The results show that our method’s errors are close to the experimental results on Modelnet40, which verifies the good generalization ability of our method. All experiments prove that the proposed method is superior to state-of-the-art PCReg algorithms in terms of accuracy and complexity.

## 1. Introduction

Point cloud registration (PCReg) refers to the problem of finding the rigid transformation that maximizes the overlap between similar sections of two or more point clouds. As a fundamental technique in 3D data processing, it is employed in many fields including computer vision, robotics, medical image analysis and computer-assisted surgery.

Researchers in the past have proposed methods [1,2,3,4,5] to address the PCReg problem. However, many of them are prone to converging to local optima. With the advent of deep neural networks (DNNs), it has been shown [6,7,8] that PCReg methods using DNNs can achieve higher accuracy and robustness to inaccurate transformation when compared to traditional methods. The learning-based PCReg method processes unordered point clouds and extracts features through a deep learning network [9,10,11]; then, the similarity of these features is used to calculate the transformation. However, most of these methods cannot cope with large transformations [7,12]; specifically, they achieve high accuracy only when the rotation and translation are limited to [−45${}^{\circ }$, 45${}^{\circ }$] and [−0.5 m, 0.5 m], respectively. Most researchers directly use the 3D coordinates of points as inputs for feature extraction. However, the values of 3D coordinates are susceptible to rigid transformation; the same point will have different features after transformation. Since they are not robust to transformation and therefore cannot act as stable inputs for DNNs, DNNs cannot learn the features with transformation invariance [13,14].

**Figure 1:**
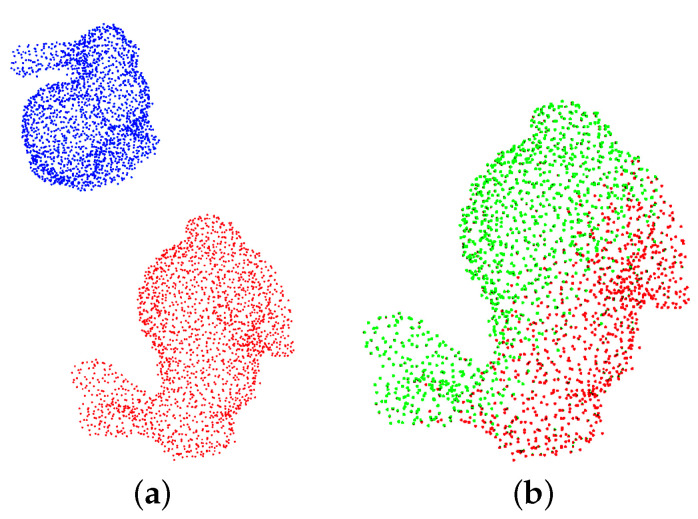
Point cloud registration with TIF-Reg. (**a**) The input point clouds. (**b**) The registered point clouds. The relative rotation of [x,y,z] is [77.4${}^{\circ }$, −129.5${}^{\circ }$, 17.5${}^{\circ }$] and the relative translation of [x,y,z] is [2.9 m, −1.0 m, −2.5 m].

In this work, we propose a novel PCReg method with rigid transform-invariant features, named TIF-Reg, to overcome the limitations of existing learning-based methods, enabling accurate PCReg with large rigid transformations by constructing transform-invariant features. Figure 1 shows the registration result of TIF-Reg. It includes four modules, which are the transform-invariant feature extraction module, deep feature embedding module, corresponding point generation module and decoupled SVD module. The transform-invariant feature extraction module constructs the transform-invariant features (TIF) based on the spatial structure of the point cloud. The deep feature embedding module embeds TIF into a high-dimensional space, leveraging mini-DGCNN to improve the expressivity of the features. The corresponding point generation module generates the corresponding points of input clouds through an attention-based module. The decoupled SVD module calculates the transformation using an SVD module. We test our method on ModelNet40 and TUM3D under various settings and compare them with traditional and learning-based methods to demonstrate the superior performance of the proposed method in terms of accuracy and complexity.

The key contributions of this work are summarized as follows:

1. We propose the leveraging of transform-invariant features in the PCReg problem and evaluate the expressivity of the features;
2. We propose a novel PCReg method that is robust to the large rigid transformation between source clouds and target clouds;
3. We evaluate the performance of our method under several settings, demonstrating the effectiveness of the proposed method.

## 2. Related Work

### 2.1. Hand-Crafted Features

In PCReg problems, 3D coordinates are commonly used to find corresponding points [3]. This is simple and effective, but these discrete points cannot holistically describe the characteristics of the point cloud and result in an inaccuracy in registration. In order to improve the effectiveness of the algorithm, researchers have attempted to extract features with a stronger representation ability. FPH [15] and FPFH [16] described the curvature of sampling points using a multi-dimensional histogram and then obtained the geometric features of the K-neighborhood of sampling points. VFH [17] extended FPFH with a viewpoint component to maintain the pose discrimination of features. CVFH [18] calculated VFH features in several point clusters to reduce the loss of key points. LOAM [19] constructed edge points and plane points by curvature and achieved good results in the continuous frame registration during SLAM. To summarize, the linear and planar features of point clouds are considered to improve registration accuracy.

### 2.2. Traditional Registration Methods

Many variations of ICP such as [20,21] have been proposed in the past several years; however, ICP and most of its variants can only produce local optimal estimates. In [22], the authors developed Go-ICP, a branch and bound-based optimization approach to obtain the globally optimal pose. In [23], the authors described the point cloud and surface normal densities by utilizing Bayesian nonparametrics to improve the robustness of registration. In [2,24], the authors attempted to identify global optima through mixed-integer programming and Riemannian optimization. The above methods generally are time-expensive and impractical for real-time systems. Ransac [25] randomly and repeatedly sampled the point cloud, calculated the rigid transformation based on FPFH and selected the optimal value. NDT [1] divided the point cloud into a certain number of grids, calculated the probability density function (PDF) of each grid according to the normal distribution and obtained the global registration result of the point cloud by matching the PDF. Recently, Refs. [26,27] formulated the point cloud registration problem in a probabilistic manner by modeling the underlying structure of the scene as a latent probability distribution and using the EM (Expectation Maximization) algorithm, respectively.

### 2.3. Deep Features Extraction Methods

Due to the fact that point clouds have no inherent order, general image feature extraction methods are not suitable for point clouds. In [28,29], the authors tried to solve this problem by voxelizing the point cloud, but this approach results in a loss of information. PointNet [11] first proposed a DNN to directly consume the original point cloud using a symmetric function. PointNet++ [9] optimized PointNet for local feature extraction and extracted different features for each point through feature interpolation. In [30], the authors used a kd-tree structure to form the computational graph and designed a kd-network with smaller memory footprints and more efficient computations compared to uniform voxel grids. In [31], the authors improved PointNet by enlarging the receptive field over the 3D scene. In [32], the authors designed a novel octree data structure to efficiently store the octant information and CNN features into the graphics memory. In [10], the authors proposed a graph neural network (GNN), establishing a neighborhood for each point and constructing a dynamic edge through the 3D coordinates of each point and finally restoring the graph structure.

### 2.4. Registration Based on Learning

After PointNet was proposed, the DNNs’ ability to extract features from point clouds was discovered, and thus many learning-based PCReg algorithms emerged. PointNetLK [6] drew on the Lucas–Kanade (LK) algorithm and Inverse Compositional (IC) formulation in 2D images and tracked the rotation and translation of the entire point cloud through iterative optimization. DCP [7] highlighted the limitations of PointNet and used DGCNN as an alternative. In addition, theat work referred to [33] in the NLP field, converted the point cloud registration problem into a seq2seq problem and finally used SVD to obtain the rotation and translation matrix. In [34], the authors realized partial-to-partial registration iteratively using an actor–critic closest point module. In [12,35], the authors achieved PCReg in autonomous driving scenarios by keypoint detection and corresponding point generation. In [36], the authors directly predicted a rigid transformation attached to each correspondence by operating on the pool of pose predictions. Generally, these methods utilize deep features learned by DNNs instead of hand-crafted features to achieve higher accuracy.

## 3. TIF-Reg Algorithm

**Figure 2:**
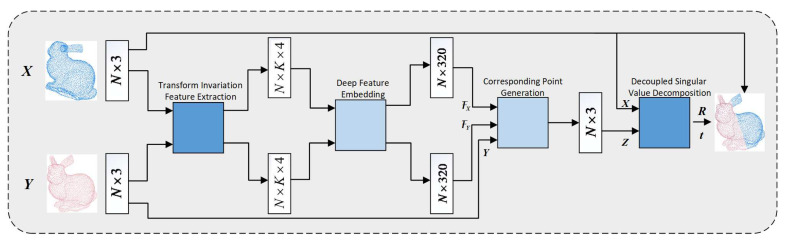
The architecture of the TIF-Reg.

The architecture of the proposed TIF-Reg is shown in Figure 2. The input includes the source point cloud *X* (blue points) and the target point cloud *Y* (red points). First, we extract TIF from the input and map TIF into high-dimensional space via DNN. Then, we generate the corresponding points using an attention mechanism. Lastly, we calculate the transformation using a decoupled SVD.

### 3.1. Transform-Invariant Feature Extraction

TIF are point cloud features that are invariant under rigid transformations of the point cloud, including rotations and translations.

**Figure 3:**
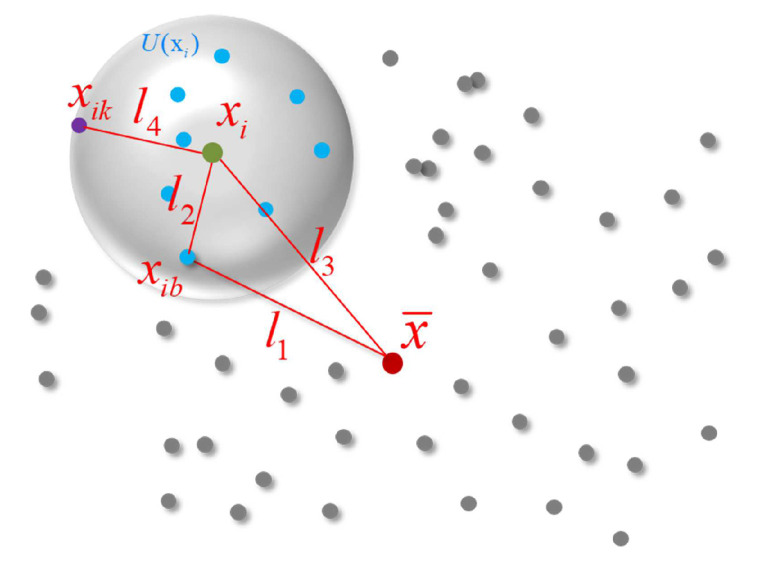
TIF. The points in blue are the points in $U(x_{i})$. The TIF of $\left(x_{ib}\right)$ is represented by Euclidean distances $l_{1},l_{2},l_{3}$ and $l_{4}$.

As shown in Figure 3, consider the point cloud with *N* points: $X=\{x_{1},x_{2},\ldots ,x_{N}\}$. For each $x_{i}\in X\left(i=1,2,\ldots ,N\right)$, we construct the neighborhood set of $x_{i}$ denoted $U(x_{i})$ through the k-nearest neighbors algorithm (K-NN). Hence, there are *N* neighborhoods in *X*, and each neighborhood contains *K* points. Each point in *X* is described as $x_{ib}\in U\left(x_{i}\right)$(b=1,2,…,k), and we define the TIF of $x_{ib}$ as

$$L_{x_{ib}}=\left[l_{1},l_{2},l_{3},l_{4}\right]$$

and

(1) $$\left\{\begin{array}{cc} l_{1}=\left|\right|x_{ib}-\bar{x}\left|\right|, & \\ l_{2}=\left|\right|x_{ib}-x_{i}\left|\right|, & \\ l_{3}=\left|\right|x_{i}-\bar{x}\left|\right|, & \\ l_{4}=\left|\right|x_{ik}-x_{i}\left|\right|. \end{array}\right.$$

where $\bar{x}$ is the center of *X* and $x_{ik}$ is the last point in $U(x_{i})$. $l_{1},l_{2},l_{3}$ form a triangular structure, which we call a triangular feature. $l_{4}$ describes the density of the k-NN to some degree and is called the local density feature. The triangular feature and local density feature represent the relative position characteristics between the points and the local distribution characteristics of the point cloud. Unlike 3D coordinates, the TIF can remain stable when point clouds are transformed. Therefore, they are more suitable for PCReg problems than 3D coordinates. In Figure 2, the input is an N×3 tensor, representing the 3D coordinates of the point cloud. After TIF extraction, the point cloud is represented as an N×K×4 tensor, where *K* refers to the points from k-NN.

#### 3.1.1. Triangular Feature

Since point clouds are sets of points without any specific order, an input cloud with *N* points can have N! permutations, making it difficult to obtain the position of a specific point [11]. However, the relative distance between points is invariant. To ensure the invariance of TIF to rigid transformation, we attempt to seek out two points with fixed relative positions (we regard them as indexable points) and define the Euclidean distance of indexable points as the descriptor of a point.

Firstly, it is easy to see that the shape distribution of *X* will not change after rigid transformation, meaning the relative position of the center $\bar{x}$ in a point cloud will remain the same after transformation. Note that we are focused on relative positions and not coordinates. Moreover, since the relative distance of points will not be affected by transformations, for each $x_{i}\in X$, the k-NN $U(x_{i})$ will be constant during transformations as well. That is, for each $x_{ij}\in U(x_{i})$, its neighborhood set center $x_{i}$ will remain stable during transformation. According to the above analyses, we offer each $x_{ij}\in U(x_{i})$ two indexable points: $\bar{x}$ and $x_{i}$. Connecting the three points together, we can obtain the triangular feature of $x_{ij}$, represented as the side length of the triangle $l_{1},l_{2},l_{3}$, as shown in (1).

**Figure 4:**
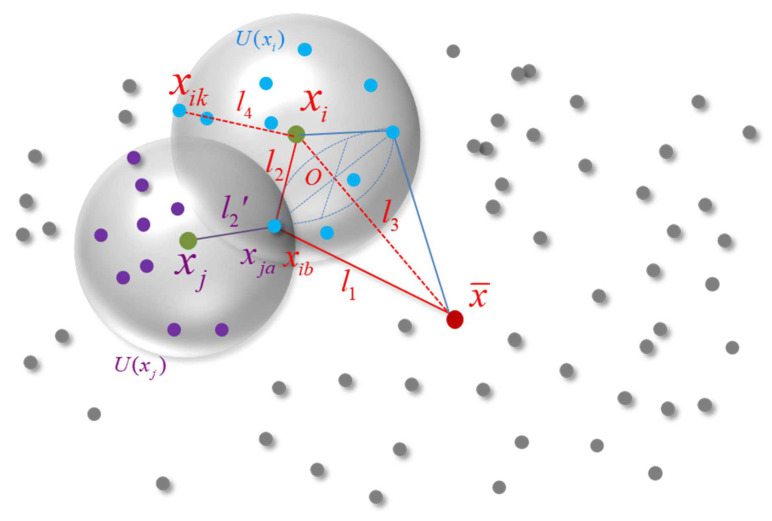
TIF analysis. $l_{3},l_{4}$ are necessary for improving the uniqueness of TIF.

$l_{3}$ may seem to offer nothing in terms of improving the representation ability of a triangular feature; however, it can be proved that the full triangular feature can be more effective than when only considering $l_{1}$ and $l_{2}$. For example, considering $x_{j}\in X$ from Figure 4, $U(x_{j})$ is the k-NN of $x_{j}$, and $x_{ja}\in U(x_{i})\;\left(a=1,2,\ldots ,k\right)$. If we only consider $l_{1}$ and $l_{2}$, then we can obtain the feature of $x_{ja}$, by calculating $l_{1}=\left|\right|x_{ja}-\bar{x}\left|\right|$, $l_{2}^{{}^{\prime }}=\left|\right|x_{ja}-x_{j}\left|\right|$. Note how the features of $x_{ib}$ and $x_{ja}$ for $x_{i}$ and $x_{j}$, respectively, are the same when $l_{2}=l_{2}^{{}^{\prime }}$, leading to the weak uniqueness of the features. What makes the situation worse is that innumerable points with the same feature can be found on the sphere with $x_{ib}$ as center and $l_{3}$ as radius. Therefore, $l_{3}$ is necessary to describe the global characteristics of point clouds and is helpful to distinguish different k-NN neighborhoods.

#### 3.1.2. Local Density Feature

Although we have built triangular features for each point, this is still insufficient for effective uniqueness in a 3D point cloud. For example, in Figure 4, if we rotate the point $x_{ij}$ with $l_{3}$ as the axis, we can obtain the circle ⊙O, and each point on ⊙O (such as $x_{in}$) has the same triangular feature as $x_{ij}$.

To overcome this issue, we take inspiration from NDT [1]. The distribution of a point cloud will not be affected by the reordering of the points in the point cloud and remains unchanged during rigid transformation. NDT places the point cloud on a grid and calculates the probability distribution function of a point that is observed in a particular unit of the grid and then performs registration using the likelihood function of the point cloud distribution. Similarly, in this work, we directly construct the local density feature of the point cloud with the k-NN as the unit. In order to avoid one of the features from being concealed due to the magnitude difference between the triangular feature and the local density feature, we must instead express the local density feature in terms of Euclidean distance. Since the number of points in $U(x_{i})$ is fixed, the radius of $U(x_{i})$ is one indicator of the density of the local point cloud. Generally, the sparser the point cloud, the larger the radius, and the denser the point cloud, the smaller the radius. Therefore, the radius of $U(x_{i})$, which can be used effectively with the triangular feature, is used in this paper to describe the local density feature of $x_{i}$.

### 3.2. Deep Feature Embedding

In Section 3.1, the original 3D points have been transformed to 4D TIF features. In this section, we embed the TIF features via a deep neural network into high-dimensional space to strengthen the representation ability of feature descriptors. Mini-DGCNN, a simplified version of DGCNN, is used here. It uses a dynamic graph structure: it constructs a local k-NN graph for each $x_{i}\in X$ and pools the features of the points in $U(x_{i})$ together using a max pooling layer.

In this work, the mini-DGCNN only utilizes a static graph in DGCNN, which helps to reduce the network complexity and yet still achieve the same performance of registration. As shown in Figure 2, the Deep Feature Embedding (DFE) layer transforms the N×K×4 feature to an N×320 tensor.

### 3.3. Corresponding Point Cloud Generation

A prominent part of the typical point cloud registration process is the construction of a matching between the points in the original and target point clouds. The ICP algorithm iteratively updates the transformation by minimizing the distance between corresponding points to gradually optimize their alignments. However, this method is prone to stalling in local optima and can lead to poor registration results. Inspired by the attention mechanism in [7,33], we propose a destination point cloud generation method based on point cloud similarity rather using a point-to-point mapping between the source and target point clouds.

The attention mechanism is derived from the study of human vision and is widely used in the natural language processing (NLP) field to handle sequence-to-sequence (seq2seq) problems, such as machine translation and question answering. During observations, in order to efficiently distribute limited attention resources, humans tend to selectively focus on the more important data or regions of the subject and ignore the less useful noise. Similarly, in seq2seq problems, researchers use the attention mechanism to select information that is critical to the task at hand from a large amount of input information. In this paper, we regard the PCReg as a seq2seq problem, with point clouds *X* and *Y* as the source and target sequence, respectively. The purpose is to generate an output destination point cloud *Z* that is as similar as possible to *Y* with a mapping to correspond each point in *X* to each point in *Z*. With this goal in mind, we apply the attention mechanism to generate *Z*.

The attention weight *W* is obtained using the similarity between features in *X* and *Y*:

(2) $$W=\mathrm{softmax}(F_{X}{F_{Y}}^{T})$$

where $F_{X}$ and $F_{Y}$ are the deep features obtained in Section 3.2 from *X* and *Y*, respectively. Then, *Z*, the corresponding point cloud of *X*, can be generated from *W* and *Y*:

(3) Z=WY

For each $x_{i}\in X\left(i=1,2,\ldots ,N\right)$, we generate its corresponding point $z_{i}\in Z$(i=1,2,…,N) using the similarity between features in *X* and *Y*. This approach avoids constructing a direct matching of points between *X* and *Y* since the rigid transformation is obtained with respect to *X* and *Z* instead of *X* and *Y*. Since *Z* has a one-to-one point correspondence with *X*, we can achieve the results in one shot, avoiding local optima during the iteration.

### 3.4. Decoupled SVD

After obtaining the optimal destination point cloud, the final step is to calculate the relative transformation between it and the original. Multilayer perceptrons (MLP) and singular value decomposition (SVD) are commonly used to compute these results, and in this work, we apply the latter as it was proven to be more effective for registration than MLP in recent work [7]. More concretely, we aim to find the transformation $\left[R_{XY},t_{XY}\right]$ between *X* and *Y* that minimizes the error *E*:

(4) $$E\left(R_{XY},t_{XY}\right)=\frac{1}{N}\sum_{i=1}^{N}\left|\right|R_{XY}x_{i}+t_{XY}-z_{i}\left|\right|$$

Here, $z_{i}\in Z$, where *Z* is calculated in the last section to replace *Y* in a one-to-one mapping. The cross-covariance matrix *H* of *X* and *Z* is

(5) $$H=\sum_{i=1}^{N}x_{i}^{\mathrm{cen}}{\left(z_{i}^{\mathrm{cen}}\right)}^{T}$$

Here, $x_{i}^{\mathrm{cen}}=x_{i}-\bar{x}$ and $z_{i}^{\mathrm{cen}}=z_{i}-\bar{z}$. $\bar{x}$ and $\bar{z}$ are the center of *X* and *Z* respectively. Define the centralized *X* and *Z* as $X_{\mathrm{cen}}$ and $Z_{\mathrm{cen}}$, then $x_{i}^{\mathrm{cen}}\in X_{\mathrm{cen}}$ (i=1,2,…,N) and $z_{i}^{\mathrm{cen}}\in Z_{\mathrm{cen}}$(i=1,2,…,N). Using SVD, the cross-covariance matrix *H* can be decomposed as

(6) $$H={USV}^{T}$$

We can use *R* and *t* to minimize (4) based on (6):

(7) $$\left\{\begin{array}{cc} R_{XY}=VU^{T}, & \\ t_{XY}=-R_{XY}\bar{x}+\bar{z}. \end{array}\right.$$

From the experimental results (see table in Section 4.2), we find that when using the original SVD, the proposed method maintains high accuracy when the rotation is within the range [−180${}^{\circ }$, 180${}^{\circ }$] and the translation is within the range [−20 m, 20 m]. However, this will gradually decrease with larger translations. In order to solve this issue, in this section, we decouple the calculation of translation and rotation by introducing a two-step method. The proposed method with original SVD will be dubbed TIF-Reg, and the proposed method with decoupled SVD will be dubbed TIF-Reg2. We discuss the details of TIF-Reg2 below.

Step 1: Calculate rotation

In step 1, instead of *X* and *Y*, we use $X_{\mathrm{cen}}$ and $Y_{\mathrm{cen}}$ as the inputs of the proposed method’s attention mechanism to generate $Z_{\mathrm{cen}}$. According to (4), the rotation between *X* and *Y* can be calculated by using only $X_{\mathrm{cen}}$ and $Z_{\mathrm{cen}}$. That is, the rotation has no relation to the translation.

$X_{\mathrm{cen}}$ will coincide completely with $Y_{\mathrm{cen}}$ only when *X* has the same distribution as *Y*; otherwise, there will be a translation $t_{l}$ between them. The greater the difference between the distributions, the greater the translation. Generally, $t_{l}$ is much smaller than $t_{XY}$, thus avoiding the previously mentioned effect of large translations on overall accuracy. In step 1, $R_{XY}$ and $t_{l}$ are calculated.

(8) $$t_{l}=-R_{XY}{\bar{x}}_{\mathrm{cen}}+{\bar{z}}_{\mathrm{cen}}$$

Here, ${\bar{z}}_{\mathrm{cen}}$ is the center of $Z_{\mathrm{cen}}$.

Step 2: Calculate translation

We first note that $t_{XY}$, the relative translation between *X* and *Y*, can be decomposed as follows, where $t_{l}$ is as defined in step 1, and $t_{g}$ is the remainder of the final translation.

(9) $$t_{XY}=t_{l}+t_{g}$$

To calculate $t_{g}$, we first transform *X* to $X^{\prime }$ using the values obtained in step 1:

(10) $$X^{\prime }=R_{XY}\bar{X}+t_{l}$$

We denote the center of $X^{\prime }$ as $\bar{x}{}^{\prime }$ and obtain $t_{g}=\bar{y}-\bar{x}{}^{\prime }$, completing our calculations for the translation, $t_{XY}$, between *X* and *Y*.

In this section, we decompose $t_{XY}$ to $t_{l}$ and $t_{g}$ by centralizing the point cloud. $t_{l}$ and $R_{XY}$ are calculated in step 1, and $t_{g}$ is calculated in step 2. This approach decouples rotation and translation and therefore increases the robustness of the proposed method to large translation.

### 3.5. Loss Function

Considering the relationship between *X* and *Y*, we have

(11) $$Y={R_{XY}X}_{l}+t_{XY}$$

Due to the lack of order of the point cloud, the difference between $Y^{gt}$ and $Y^{pre}$ cannot be calculated directly, where gt represents the ground truth value (referring to the actual target point cloud) and pre represents the predicted value (referring to the destination point cloud obtained by the algorithm). Instead, we represent the difference using the loss function:

(12) $$Loss=\left|\right|{R_{XY}^{pre}}^{T}R_{XY}^{gt}-{I||}^{2}+\left|\right|t_{XY}^{pre}-t_{XY}^{gt}{\left|\right|}^{2}$$

## 4. Experiments

The proposed method TIF-Reg was evaluated against ModelNet40 [29] and TUM 3D object (TUM3D) [37] datasets. All experiments were performed on a laptop computer with an Intel I7-8750 CPU, an Nvidia GTX 1060 GPU and with 24 GB RAM.

Implementation Details of TIF-Reg: The architecture of the TIF-Reg is shown in Figure 2. In the deep feature embedding module, the EdgeConv (denoted as DGCNN [10]) layers were used in mini-DGCNN and the numbers of filters in each layer were 64, 64, 64, 128 and 320. The optimizer applied here was Adam with an initial learning rate of 0.0001, which was divided by 10 at epochs 40 and 60. The total training epochs were 80 and the training took approximately 4 h in our condition.

Baselines: We used five baselines including the traditional methods ICP [3] , GO-ICP [22] and Ransac+ICP, and learning-based methods PointnetLK [6] and DCP-v2 [7] (referred to as DCP [7]).

Evaluation metrics: We measured the root mean squared error (RMSE) and mean absolute error (MAE) between the ground truth value and predicted value for both rotation (*R*) and translation (*t*), which are represented as RMSE(R), RMSE(t), AME(R) and AME(t), respectively. The metrics related to rotation are in units of degrees. The metrics related to translation are in units of meters.

ModelNet40 dataset: This dataset consists of 12,311 CAD models from 40 categories. We randomly sampled 2048 points from the mesh faces and rescaled points into a unit sphere. In our experiments, we split up each category randomly, obtaining 9843 models for training and 2468 models for testing. For each model, 1024 points from the outer surface were uniformly sampled, and all of the points were centered and rescaled to fit in the unit sphere.

TUM3D: This dataset includes 20 CAD models from 20 different categories of 3D point cloud models and is significantly different from the ModelNet40. We used all of the 3D models for testing. For each model, 4096 points were uniformly sampled from the original CAD and all of the points were rescaled to fit in the unit sphere.

### 4.1. Train and Test on ModelNet40

Firstly, we trained the learning-based methods on the first 20 categories and tested all of the PCReg methods on the same 20 categories as well. We took the sampled point cloud from CAD as target *Y*. *X* was obtained through an arbitrary transformation of *Y*. where the rotation was in the range [−45${}^{\circ }$, 45${}^{\circ }$] and the translation was in the range [−0.5 m, 0.5 m].

Table 1 shows the results of this experiment. We can see that ICP had the largest errors (the RMSE and MAE) both in rotation and translation, while the traditional algorithm Go-ICP achieved similar results to the PointNetLK algorithm based on the deep neural network. Ransac+ICP achieved the middle performance for all methods but performed best out of the traditional methods. Both DCP and TIF-Reg had lower errors, but TIF-Reg performed the best and outperformed other methods by roughly an order of magnitude.

We tested the generalizability of the different methods using different categories for training and testing. Learning-based methods were trained on the first 20 categories and tested on the last 20 categories. Traditional methods, which do not require the training of the model, were also tested on the last 20 categories.

As shown in Table 2, ICP still had the largest error while GO-ICP and Ransac+ICP had similar errors, but Ransac+ICP achieved a much better result than the previous experiment. TIF-Reg still exhibited the best performance among all the methods. In this experiment, almost all methods’ accuracies declined to varying degrees, except Ransac+ICP and TIF-REg. This shows that the methods DCP and PointNetLK based on deep learning achieved a slightly poor generalization of data in different categories, but our method was basically unaffected by different data categories.

### 4.2. Robustness to Transformation (Rotation and Translation)

This experiment tested the robustness of TIF-Reg to transformation, which is essential for evaluating the effectiveness of the PCReg method. This experiment was divided into two steps: first, we kept the translation within [−0.5 m, 0.5 m] while gradually expanding the rotation from [−45${}^{\circ }$, 45${}^{\circ }$] to [−180${}^{\circ }$, 180${}^{\circ }$] to test the robustness to rotation. Then, we kept the rotation within [−180${}^{\circ }$, 180${}^{\circ }$] while gradually expanding the translation from [−2 m, 2 m] to [−20 m, 20 m] to test the robustness to translation. In this section, learning-based methods were trained on the first 20 categories and tested on the last 20 categories.

Table 3 shows the rotation robustness of all methods (see Table 2 for rotation within [−45${}^{\circ }$, 45${}^{\circ }$]. Table 4 shows the translation robustness. According to Table 3 and Table 4, ICP, GO-ICP and PointNetLK almost failed under larger rotation and translation. DCP was no longer valid under lager translation.The performance of Ransac+ICP was much better than the above methods, but compared with the first two experiments, the error was still large under larger rotation and translation. Of all methods, TIF-Reg demonstrated the highest robustness to transformation throughout the experiment. As the angle of rotation increased, the accuracy of TIF-Reg decreased slightly, but it had the lowest error and was the most stable.

### 4.3. Effectiveness of TIF

In this experiment, in order to verify the effectiveness of TIF, we compared the performance of the proposed method when using 3D coordinates, an incomplete TIF (only three of $l_{1},l_{2},l_{3},l_{4}$ are selected) and the complete TIF. The training set and test set used here were the same as Section 4.2, and the random transformation was within [−180${}^{\circ }$, 180${}^{\circ }$] and [−8 m, 8 m].

As shown in Table 5, the algorithm failed when using 3D coordinates. It performed well when using an incomplete TIF; however, the best results occurred when using the complete TIF. This demonstrates the effectiveness of not only TIF in the PCReg problem but also of each individual element in TIF in improving its representation ability.

### 4.4. Robustness to Large Translation

We had already tested the translation robustness of the proposed method in Section 4.2, but in this experiment, we tested the proposed method’s performance with regard to even larger translations. The dataset used here was the same as Section 4.2, the rotation was within [−180${}^{\circ }$, 180${}^{\circ }$], and the translation was expanded from [−20 m, 20 m] to [−120 m, 120 m].

**Figure 5:**
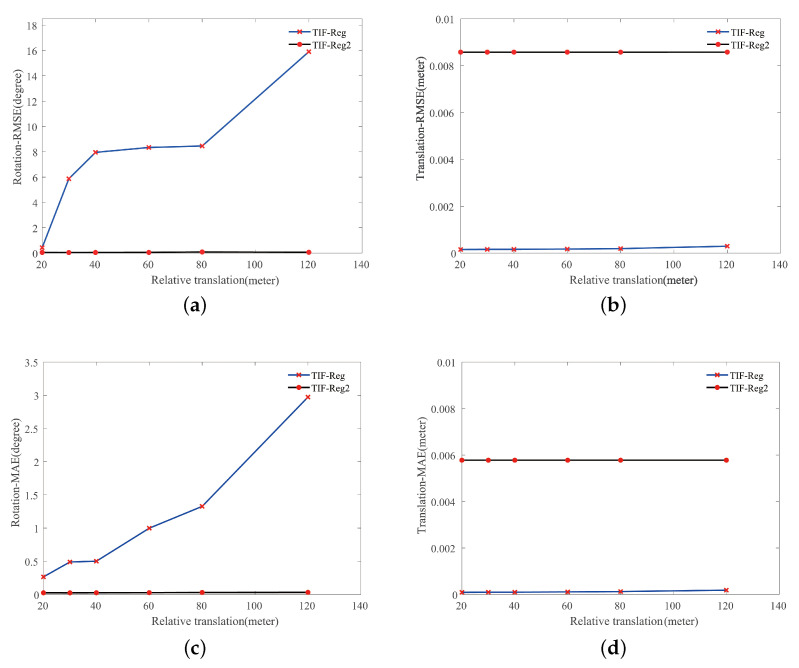
The results of TIF-Reg and TIF-Reg2 in response to large translations. (**a**) The results for the RMSE of rotation. (**b**) The results for the RMSE of translation. (**c**) The results for the MAE of rotation. (**d**) The results for the MAE of translation.

Figure 5 displays the error of TIF-Reg and TIF-Reg2 with large translations. According to Figure 5a,c, as the translation increased, the rotation error of TIF-Reg increased significantly, while TIF-Reg2 maintained high precision. Figure 5b,d demonstrates that both TIF-Reg and TIF-Reg2 hardly increased in terms of translation error and maintained an error of less than 0.01. The error of TIF-Reg was slightly lower than that of TIF-Reg2. This shows that the performance of the decoupled SVD module is superior to that of using SVD directly.

### 4.5. Generalization on New Test Set

In this experiment, in order to further test the generalization of the proposed method, we used the new dataset TUM3D. We randomly performed 36 transformations on each of the 20 sampled CAD models to produce 720 source point clouds for the test set. The settings here were the same as Section 4.2 except for the test set.

The experiment result was showed in Table 6. Here, there are two arguments R and t and Table 6 only shows t. The first line of Table 6 shows R in the range of [−45${}^{\circ }$, 45${}^{\circ }$], the second line shows R in the range of [−90${}^{\circ }$, 90${}^{\circ }$], and the other lines shows *R* R in the range of [−180${}^{\circ }$, 180${}^{\circ }$]. The table shows that the TIF-Reg model trained on ModelNet40 was still able to maintain a high accuracy on the TUM3D dataset and had strong robustness to transformation as well.

### 4.6. Complexity

This experiment was used to compare the complexity of the algorithm, including time complexity and model complexity. The complexity of the algorithm involves many factors, such as computation, real-time performance and hardware costs.

#### 4.6.1. Time Complexity

We profiled the inference time of different methods in this experiment. In order to make the comparison more comprehensive, we tested the time complexity with point clouds of different sizes. The inference time was measured in seconds. Note that Go-ICP was ignored in the experiment as it took over 16 s, far exceeding other methods.

As shown in Table 7, the time complexity of Ransac+ICP was the highest, and it was less affected by the number of points than other methods. The time complexity of two deep learning-based methods, PointNetLK and DCP, was most affected by the number of point clouds. As the number of point clouds increased, so did their time complexity. TIF-Reg showed the best real-time performance among learning-based methods and was equivalent to ICP, which was the best of the baselines (the blue line of TIF-Reg covers the black line of ICP, and the “O” markings of ICP cover the “X” markings of TIF-Reg).

#### 4.6.2. Model Complexity

Since the traditional methods (ICP, Ransac+ICP, Go-ICP) do not have models, only learning-based methods (PointnetLK, DCP, TIF-Reg) were compared in the experiment. As shown in Table 8, the TIF-Reg model occupied the least space.This shows that the calculation process involved in our method is the most simple compared with the other two methods using neural networks.

## 5. Discussion

In this section, we discuss the experimental results of TIF-Reg with that of other methods we used as the baselines.

### 5.1. Algorithm Accuracy

ICP [3] is an algorithm that easily falls into local optimal solutions, and the key to its success is a good initial transformation. It is obvious that ICP cannot deal with situations with large transformations. As we can see from Table 2, Table 3 and Table 4, the errors of ICP become larger as the transformation becomes larger. Go-ICP [22] improved ICP by introducing branch-and-bound (BnB) to improve the global search ability of the algorithm, so it performs better than ICP, but its performance is still limited under large transformations. Table 3 and Table 4 show that when the transformation is large, Go-ICP no longer presents advantages, and its results are basically close to the ICP results. For Ransac+ICP, we used the Ransac algorithm for rough registration and ICP algorithm for fine registration. Therefore, Ransac can provide an initial transformation close to the optimal solution for ICP; thus, it can achieve higher precision. Table 2, Table 3 and Table 4 show that Ransac+ICP is the most effective method other than our method for both small and large transformations. PointNetLK [6] uses PointNet [11] to provide deep features of point clouds, but the network structure of PointNet is simple and it loses local information of point clouds, so it cannot describe point clouds well. Table 2, Table 3 and Table 4 show that its errors increase as the transformation increases. In contrast, DCP [7] and TIF-Reg use DGCNN, which can extract richer point clouds information to obtain deep features, so their features have better performance. TIF-Reg further uses TIF rather than the 3D coordinates of point clouds as the network’s input; thus, its model can deal with the point cloud registration under large translations and has better performance than DCP. We can see that the performance of TIF-reg is very little affected by large rotation or translation in Table 2, Table 3 and Table 4, and it achieves good generalization in different datasets, as can be seen in Table 6. We can also see that the use of the decoupled SVD module can further improve the performance of the algorithm under large transformations in Figure 5.

### 5.2. Algorithm Complexity

The results of algorithm complexity can be seen in Table 7. ICP [3] is the simplest traditional iterative algorithm, and its time complexity is close to TIF-Reg. When the number of points is 1024, ICP and TIF-Reg only use 0.01549 s and 0.01553 s, respectively. GO-ICP [22] introduces BnB to search for the global optimal solution; based on the iterative process of ICP, it adds the step of searching the nearest neighbor points, so its time complexity is higher than ICP. For Ransac+ICP, in order to obtain more accurate solutions, Ransac needs to carry out a large number of iterations, which greatly increases the time complexity of the algorithm. It is the most time-consuming algorithm of all methods we compared expect GO-ICP. When the number of points is 1024, the elapsed time is 0.03583 s, which is more than twice that of TIF-Reg. For the methods based on deep learning, it is well known that the larger the amount of input data of the neural network, the higher the operation cost of the network. PointnetLK [6] involves the iterative process of neural networks, while DCP [7] includes complex network structures. Both of these will necessarily increase the time complexity of the model as the data increases. When the number of points is 1024, they consume 0.08846 s and 0.27933 s, respectively. When the number of points is 4096, their time consumption becomes very close and reaches 1.16 s approximately. Our method avoids the iterative process and does not involve a complex network structure, so it can achieve the best real-time performance.

Based on the analysis above, TIF-Reg is an algorithm that ensures high accuracy while having low time consumption.

## 6. Conclusions

We have presented TIF-Reg, a novel point cloud registration approach for adapting transform-invariant features. By constructing transform-invariant features, the proposed method achieves the high-precision registration of point clouds when the rotation is within the range of [−180${}^{\circ }$, 180${}^{\circ }$] and the translation is within the range of [−20 m, 20 m]. Moreover, the proposed method is almost unaffected by translation due to the decoupling of translation and rotation in SVD. Experiments have shown that TIF outperforms state-of-the-art methods in many aspects, including accuracy, robustness and complexity. Its considerable potential in many applications allows TIF to be easily integrated into other networks. Finally, we believe that our work presents an important step forward for the community as it affords an effective strategy for the point cloud registration framework, as well as presenting an innovation in deep feature extraction for all deep learning networks.

## Figures and Tables

**Table 1:** Training and testing on same categories with ModelNet40.

Model	RMSE(R)	MAE(R)	RMSE(t)	MAE(t)
ICP	24.671776	19.745914	0.272832	0.232474
GO-ICP	12.540327	2.662239	0.022866	0.007269
Ransac+ICP	5.796506	0.571111	0.002664	0.000620
PointNetLK	15.00359	4.623225	0.0294655	0.007932
DCP	1.110271	0.750817	0.001732	0.001193
TIF-Reg (ours)	0.032146	0.018655	0.000161	0.000086

**Table 2:** Test on different categories in ModelNet40.

Model	RMSE(R)	MAE(R)	RMSE(t)	MAE(t)
ICP	25.776054	21.047745	0.275492	0.235166
GO-ICP	15.169330	3.020630	0.024685	0.006873
Ransac+ICP	2.092478	0.079216	0.002046	0.000086
PointNetLK	17.138983	5.858677	0.034564	0.010009
DCP	3.516694	2.452658	0.019696	0.015069
TIF-Reg (ours)	0.026178	0.016377	0.000152	0.000089

**Table 3:** Testing rotation robustness with translation within [−0.5 m, 0.5 m].

	[−90${}^{\circ }$, 90${}^{\circ }$]		[−180${}^{\circ }$, 180${}^{\circ }$]	
Method	RMSE(R)	RMSE(t)	RMSE(R)	RMSE(t)
ICP	50.779556	0.279689	103.976852	0.278700
GO-ICP	55.838398	0.051618	98.706337	0.071191
Ransac+ICP	8.590699	0.008878	11.678988	0.005560
PointNetLK	34.286675	0.043960	84.266951	0.066225
DCP	14.976441	0.023615	50.218693	0.028399
TIF-Reg (ours)	0.052140	0.000152	0.048890	0.000152

**Table 4:** Testing translation robustness with rotation within [−180${}^{\circ }$, 180${}^{\circ }$].

	[−2 m, 2 m]		[−8 m, 8 m]		[−16 m, 16 m]		[−20 m, 20 m]	
Method	RMSE(R)	RMSE(t)	RMSE(R)	RMSE(t)	RMSE(R)	RMSE(t)	RMSE(R)	RMSE(t)
ICP	103.257759	1.151752	103.534096	4.614248	103.485107	9.228573	103.257805	11.535716
GO-ICP	99.908691	0.091628	107.544991	0.058547	105.837303	0.048313	105.370491	0.064317
Ransac+ICP	13.933655	0.007383	15.290869	0.125023	12.971705	0.011452	14.138081	0.009134
PointNetLK	90.978516	0.127608	93.810654	0.16224849	94.534180	0.150836	93.385742	0.170027
DCP	52.990562	0.053355	65.167519	0.022785	65.984886	0.012464	65.332367	0.010580
TIF-Reg (ours)	0.055016	0.000148	0.152886	0.000153	0.323746	0.000154	0.431104	0.000154

**Table 5:** Test results on the effectiveness of TIF.

Model	RMSE(R)	MAE(R)	RMSE(t)	MAE(t)
Coordinates	24.671776	19.745914	0.272832	0.232474
$l_{2}l_{3}l_{4}$	12.540327	2.662239	0.022866	0.007269
$l_{1}l_{3}l_{4}$	5.796506	0.571111	0.002664	0.000620
$l_{1}l_{2}l_{4}$	15.00359	4.623225	0.0294655	0.007932
$l_{1}l_{2}l_{3}$	1.110271	0.750817	0.001732	0.001193
$l_{1}l_{2}l_{3}l_{4}$	0.032146	0.018655	0.000161	0.000086

**Table 6:** Test results on TUM3D.

Model	RMSE(R)	MAE(R)	RMSE(t)	MAE(t)
[−0.5 m, 0.5 m]	0.091567	0.055786	0.000469	0.000301
[−0.5 m, 0.5 m]	0.141789	0.077974	0.000469	0.000298
[−0.5 m, 0.5 m]	0.161362	0.082230	0.000469	0.000301
[−2 m, 2 m]	0.161990	0.082795	0.000470	0.000301
[−8 m, 8 m]	0.172049	0.092861	0.000470	0.000304
[−16 m, 16 m]	0.232689	0.116418	0.000474	0.000308
[−20 m, 20 m]	0.437807	0.232852	0.001016	0.000567

**Table 7:** Time complexity.

	Method	ICP	Ransac+ICP	PointNetLK	DCP	TIF-Reg (Ours)
Number of Points						
512		0.00289	0.00709	0.01903	0.04866	0.00288
1024		0.01549	0.03583	0.08846	0.27933	0.01553
2048		0.08757	0.14639	0.40375	0.84061	0.08754
4096		0.89991	0.84154	1.17099	1.16391	0.89993

**Table 8:** Model complexity.

Model	PointNetLK	DCP	TIF-Reg (ours)
Model Size	621.9 KB	22.4 MB	495.9 KB

## Data Availability

Not applicable.
